# The Eccentric:Concentric Strength Ratio of Human Skeletal Muscle In Vivo: Meta-analysis of the Influences of Sex, Age, Joint Action, and Velocity

**DOI:** 10.1007/s40279-023-01851-y

**Published:** 2023-05-02

**Authors:** James L. Nuzzo, Matheus D. Pinto, Kazunori Nosaka, James Steele

**Affiliations:** 1 Centre for Human Performance, School of Medical and Health Sciences, Edith Cowan University, 270 Joondalup Drive, Joondalup, WA 6027 Australia; 2 School of Sport, Health, and Social Sciences, Solent University, Southampton, UK

## Abstract

For decades, researchers have observed that eccentric (ECC) muscle strength is greater than concentric (CON) muscle strength. However, knowledge of the ECC:CON strength ratio is incomplete and might inform resistance exercise prescriptions. Our purposes were to determine the magnitude of the ECC:CON ratio of human skeletal muscle in vivo and explore if sex, age, joint actions/exercises, and movement velocity impact it. A total of 340 studies were identified through searches. It was possible to analyse 1516 ECC:CON ratios, aggregated from 12,546 individuals who made up 564 groups in 335 of the identified studies. Approximately 98% of measurements occurred on isokinetic machines. Bayesian meta-analyses were performed using log-ratios as response variables then exponentiated back to raw ratios. The overall main model estimate for the ECC:CON ratio was 1.41 (95% credible interval [CI] 1.38–1.44). The ECC:CON ratio was slightly less in men (1.38 [CI 1.34–1.41]) than women (1.47 [CI 1.43–1.51]), and greater in older adults (1.62 [CI 1.57–1.68]) than younger adults (1.39 [CI 1.36–1.42]). The ratio was similar between grouped upper-body (1.42 [CI 1.38–1.46]) and lower-body joint actions/exercises (1.40 [CI 1.37–1.44]). However, heterogeneity in the ratio existed across joint actions/exercises, with point estimates ranging from 1.32 to 2.61. The ECC:CON ratio was most greatly impacted by movement velocity, with a 0.20% increase in the ratio for every 1°/s increase in velocity. The results show that ECC muscle strength is ~ 40% greater than CON muscle strength. However, the ECC:CON ratio is greatly affected by movement velocity and to lesser extents age and sex. Differences between joint actions/exercises likely exist, but more data are needed to provide more precise estimates.

## Key Points

**Table:** 

The eccentric:concentric (ECC:CON) muscle strength ratio was 1.41 when data from 12,546 individuals in 335 studies were aggregated.
Ratios were greater in older adults than younger adults (1.62 vs 1.39) and greater in women than men (1.47 vs 1.38).
Ratios were not different between upper- and lower-body joint actions/exercises (1.42 vs 1.40), but heterogeneity in ratios existed across specific joint actions/exercises.
The ECC:CON ratio was most greatly impacted by movement velocity, with a 0.20% increase in the ratio for every 1°/s increase in velocity.
Knowledge of ECC:CON muscle strength ratios can guide ECC overload prescriptions, but more data are needed to establish precise estimates across certain joint actions/exercises.

## Introduction

A repetition of a resistance exercise usually involves both an active muscle shortening phase (concentric [CON]) and an active muscle lengthening phase (eccentric [ECC]). For several decades, researchers have reported that volitional forces of human skeletal muscle in vivo are greater during the ECC than CON phase [1–3]. However, the magnitude of this difference, which is often reported as the ECC:CON strength ratio, is not entirely clear, as it might be impacted by factors such as sex [4–7], age [7], injury [8, 9], muscle group [5, 6], and movement velocity [4, 5]. In one study, Colliander and Tesch [4] submitted 27 healthy men and 13 healthy women to maximal strength testing on an isokinetic dynamometer and found that the ECC:CON strength ratio was greater in women than men (1.74 vs 1.40), the quadriceps than hamstrings (1.35 vs 1.10), and at faster than slower movement velocities (2.01 vs 1.35). Hollander et al. [6] reported somewhat similar results when measuring the ECC:CON strength ratio with the one repetition maximum (1RM). In their study, the ECC:CON strength ratio for the leg curl was 1.83 for women and 1.30 in men [6]. Moreover, across the six exercises that they assessed, the ECC:CON strength ratio ranged from 1.57 to 2.87 in women and 1.30 to 1.51 in men [6].

Though differences between ECC and CON muscle strength have been observed in human appendicular muscles since at least the 1960s [1–3], a meta-analysis on the ECC:CON strength ratio, and the factors that impact it, appears lacking. Knowledge of this ratio might have implications for the way resistance exercise is prescribed. In recent years, researchers and practitioners have expressed great interest in accentuated ECC and ECC-only resistance exercise. A number of reviews on ECC resistance exercise have been published in sports science journals [10–18], and 75–95% of strength and conditioning coaches say they prescribe ECC resistance exercise [19–21]. Moreover, new resistance exercise technologies [17, 22] have potential to deliver accentuated ECC loads in a way that is more feasible than with free weights, plate-loaded machines, and weight stack machines—the equipment most commonly used by coaches to deliver ECC overload [19, 20, 23]. Nevertheless, practitioners [20] and researchers [24–33] prescribe a wide range of relative loads for accentuated ECC and ECC-only resistance exercise (usually between 105 and 150% of the CON 1RM), and there is no consensus on the magnitude of ECC overload that should be prescribed and whether factors such as muscle group, sex, and age should be considered. Thus, meta-analysis of the magnitude of the ECC:CON strength ratio, and the factors that impact it, could help to inform and optimize delivery of ECC overload for specific exercises and populations, particularly as exercise technology continues to evolve to make accentuated ECC exercise safer and more feasible. Therefore, the purpose of this meta-analysis was to determine how much stronger skeletal muscles are during ECC than CON muscle actions. Specifically, we examined the extent to which sex, age, joint actions/exercises, and movement velocity impact the ECC:CON strength ratio of human skeletal muscle in vivo. These moderators were tested to provide more specific guidance to exercise practitioners on factors that warrant consideration for ECC overload prescriptions.

## Methods

### Literature Search

**Fig. 1:**
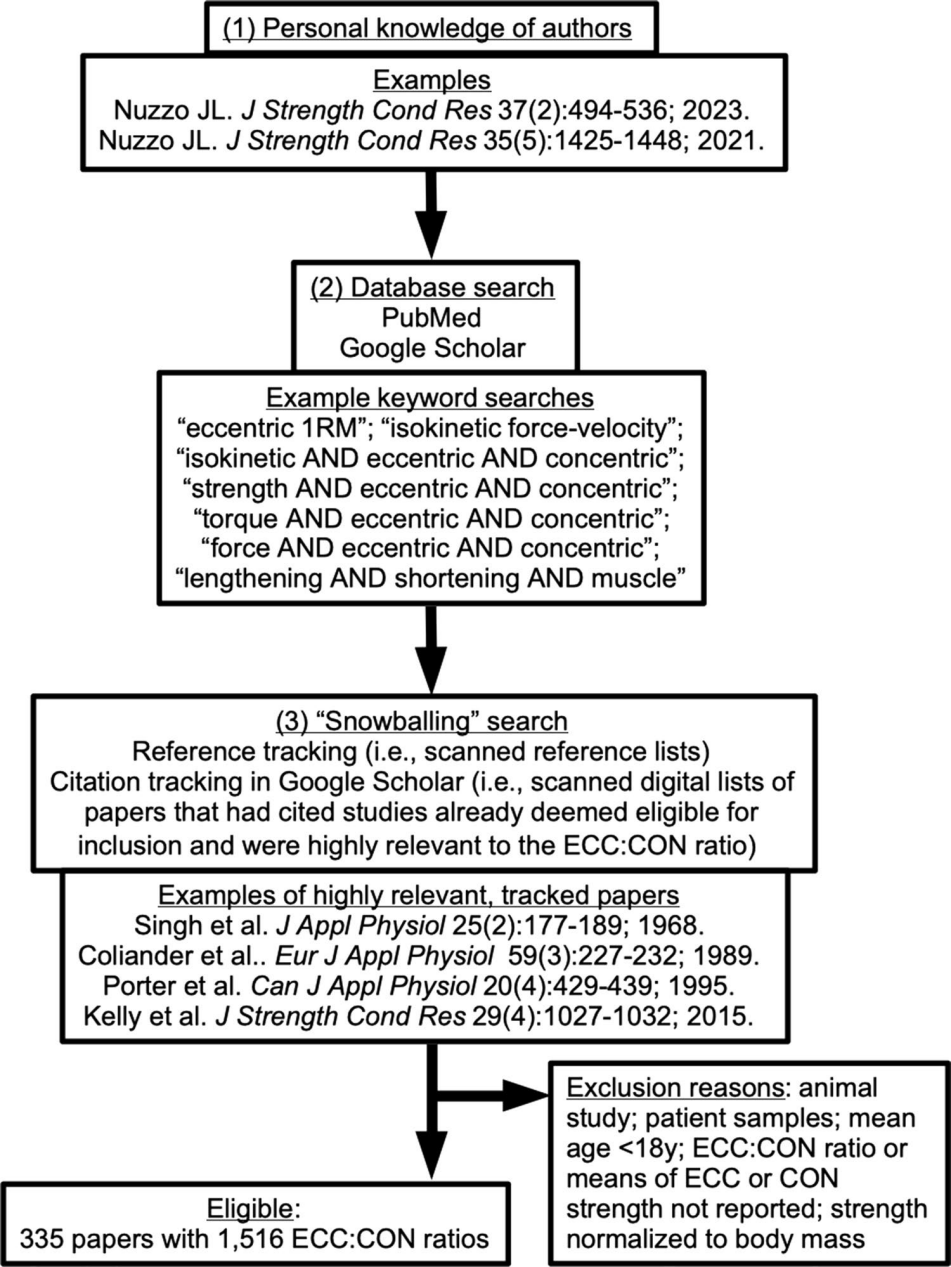
Flow diagram of search strategy. *1RM* 1 repetition maximum, *CON* concentric, *ECC* eccentric

To determine the extent to which ECC and CON muscle strength differ, we first searched for relevant literature. The search was thorough, but not necessarily systematic. We used a mixed approach similar to that described by Greenhalgh and Peacock [34]. The approach relied on the investigators’ personal knowledge from previous research [22, 35], checking of personal digital files, relevant keyword searches in PubMed and Google Scholar, and ‘snowballing’ strategies (i.e. reference and citation tracking). A flow diagram of the search strategy is presented in Fig. 1, including examples of the keyword searches. The searches were performed between May and July 2022, but were otherwise not limited by publication date.

### Eligibility

A paper was eligible for inclusion into the meta-analysis if the following conditions were met: (1) data were collected in human participants; (2) data were acquired during volitional strength tests; (3) participants were apparently healthy; (4) the mean age of participants was ≥ 18 years; (5) means of ECC and CON strength, or the ECC:CON ratio, were reported; and (6) strength data were reported in absolute units rather than body mass-normalized units. Also, for studies that involved use of isokinetic dynamometry, only studies that obtained both the ECC and CON strength measurements from the same test velocities were included. Both cross-sectional and exercise training studies were eligible for inclusion into the meta-analysis.

### Data Extraction

The data extracted from papers included sample size, number of study groups, study type (non-training or training study), sex, age group, joint actions/exercises, movement velocity, and means and standard deviations (SDs) of the ECC:CON strength ratios or the ECC and CON strength values. For age categorization, if the mean age of a study group was 18–59 years then the group was classified as ‘younger adults’. If the mean age was ≥ 60 years then the group was classified as ‘older adults’. Younger adult groups sometimes comprised competitive athletes.

In instances of unilateral strength assessments where data were available from both the right and left limbs, the data extracted from the paper were from the right limb. In instances of unilateral assessments where data were available from both the dominant and non-dominant limbs, the data extracted were from the dominant limb. For isokinetic strength tests, peak torques were always extracted instead of average torques. However, if a study reported only average torques, then average torques were extracted. With training studies, baseline strength scores were extracted for each group. Finally, for papers in which data were presented in figures, muscle strength values were estimated using a graph digitizer (WebPlotDigitizer, https://apps.automeris.io/wpd/).

### Statistical Analyses

All analysis code utilized is presented in the electronic supplementary materials (https://osf.io/8vt9h/). Given the aim of this research, we opted to take an estimation-based approach [36], based within a Bayesian framework [37]. For all analyses, effect estimates and their precision, along with conclusions based upon them, were interpreted continuously and probabilistically, considering data quality, plausibility of effect, and previous literature, all within the context of each outcome [38]. The main exploratory meta-analysis was performed using the ‘brms’ package [39] with posterior draws for visualization taken using ‘tidybayes’ [40] and ‘emmeans’ [41], and effect sizes calculated using the ‘metafor’ package [42] in R (v 4.1.2; R Core Team, https://www.r-project.org/) and RStudio (v 2022.02.03 + 492, RStudio Team, https://www.rstudio.com/). All data visualizations were made using ‘ggplot2’ [43] and ‘patchwork’ [44]. Tables were produced using ‘formattable’ [45].

We were interested in estimating the ECC:CON strength ratio; thus the log ratio was used as our effect size measure for modelling purposes. We calculated this for correlated study designs as per Lajeunesse [46], assuming a reasonable correlation of 0.7, which is not dissimilar from empirical reports of the ECC and CON strength relationship [47]. However, as both mean and variance information were not available for both ECC and CON strength for all studies, we used the recently described method of imputing the average coefficient of variation across all studies to calculate the log ratio variance [48]. When only the mean of the ratio and its variance were reported in the original study, we used the log transformed mean [49].

As the included studies often had multiple groups/conditions, and reported multiple strength measures within these, the data had a nested structure. Therefore, multilevel mixed-effects meta-analyses were performed with both inter-study and intra-study groups included as nested random intercepts in the model. Effects were weighted by inverse sampling variance to account for the within- and between-study variance. A main model included all ratios reported for all groups in each study. We conducted meta-regression and sub-group analyses of moderators (i.e. predictors of effects). Moderators examined included participant sex (men vs women), age (younger adults vs older adults), upper- vs lower-body joint actions/exercises, and velocity of movement.

For velocity of movement, we limited this to studies reporting velocity in degrees (°)/s, as this constituted the majority of observed effects.

The upper-body group consisted of the shoulder, elbow, and wrist. The lower-body group consisted of the hip, knee, and ankle, with the trunk excluded. Additional exploratory models of specific joint actions/exercises were also performed. This exploratory model included velocity and age (grand mean centred) as a fixed effect to adjust for the fact that some joints only had low numbers of effects at specific velocities or were from studies in one age group, and we anticipated that both velocity and age would impact the ECC:CON ratio.

For all models, we used uninformed priors (due to the number of effects we anticipated that the likelihood would overwhelm posterior estimates) and 23^1^ Monte Carlo Markov Chains with 2000 warmup and 6000 sampling iterations. All models had an $\widehat{R}$ value of 1.00, and trace plots were produced to visually examine chain convergence along with posterior predictive checks, which are included in the supplementary materials (https://osf.io/y7ndz). Draws were taken from the posterior distributions to calculate the mean and 95% quantile interval (referred to as the ‘credible’ interval [CI]) for each parameter estimate. These gave us the most probable value of the parameter, in addition to the range from the 2.5% to the 97.5% percentiles. We also constructed 95% prediction intervals for the main model. Log ratios were transformed back to the raw ratio scale for reporting in all instances.

## Results

A total of 340 studies were identified (see Electronic Supplementary File 1 for list of studies). Nevertheless, not all identified studies were included in the meta-analyses because effect sizes could not be calculated when only mean ratios without variances were reported. It was possible to include the results from 335 studies in our analyses. As such, the summary table of model estimates notes the number of effects, studies, and groups within studies for each estimate (Table 1). The earliest study was published in 1965 and the latest in 2023. The studies identified included 12,582 (12,546 included in analyses) participants from 575 (564 included in analyses) separate study groups, with a median sample size of 15 (range 2–734). Some studies did not report sex or age. However, 15% of the ratios extracted were from both men and women combined, 59% were from only men, and 22% were from only women. A total of 88% of the extracted ratios came from only younger adults, 10% came from only older adults, and 0.3% came from both younger and older adults. Some studies reported that participants were either competitive or recreational athletes, with 25% and 5% of the extracted ratios coming from either population, respectively. The most common sports were soccer and rugby, representing 38% and 7% of the competitive athlete groups, respectively. The proportion of studies that involved exercise training interventions was 19%. The vast majority of extracted ratios (98%) were from ECC and CON strength measured using isokinetic dynamometry, and of these, the majority were peak torques (91%). The velocities used in these isokinetic assessments ranged from 2 to 360°/s.

**Table 1:** Summary of eccentric:concentric strength ratios from all meta-analysis models

Model	Estimate	Lower CI	Upper CI	No. effects	No. studies	No. groups
Overall pooled	1.41	1.38	1.44	1516	335	564
Sex				1237	245	440
Women	1.47	1.43	1.51			
Men	1.38	1.34	1.41			
Age (years)				1488	331	556
< 60	1.39	1.36	1.42			
≥ 60	1.62	1.57	1.68			
Joint action/exercise				1469	320	543
Lower-body	1.40	1.37	1.44			
Upper-body	1.42	1.38	1.46			
Velocity (°/s)				1428	297	514
30	1.26	1.24	1.29			
60	1.34	1.31	1.37			
90	1.43	1.40	1.46			
120	1.51	1.48	1.55			
150	1.61	1.57	1.65			
180	1.71	1.67	1.75			
210	1.82	1.77	1.86			
240	1.93	1.88	1.98			
270	2.05	2.00	2.11			
300	2.18	2.12	2.24			
330	2.32	2.25	2.39			
360	2.46	2.38	2.54			

*CI* credible interval

### Main Model

**Fig. 2:**
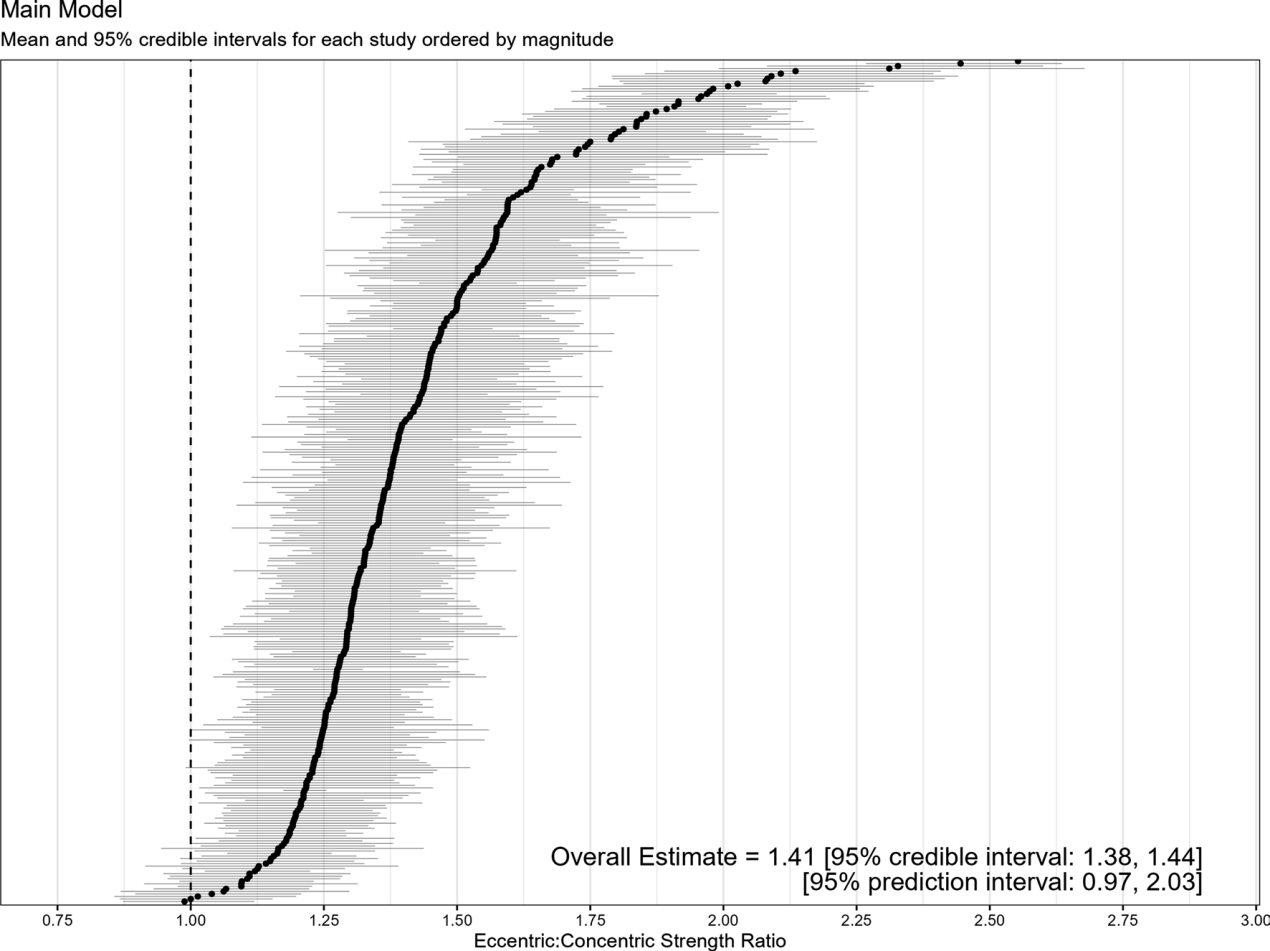
Means and 95% credible intervals of all study level estimates for eccentric:concentric strength ratios (*k* = 335)

The overall estimate from the main model revealed an ECC:CON ratio of 1.41 with CIs suggesting that the parameter value lay between 1.38 and 1.44 with 95% probability. Prediction intervals were wide, suggesting between-effect heterogeneity, with most of this variance being accounted for at the study level (see https://osf.io/ag83u). Figure 2 displays the model mean and interval estimates for each study in addition to the overall estimates and prediction interval.

### Moderators

**Fig. 3:**
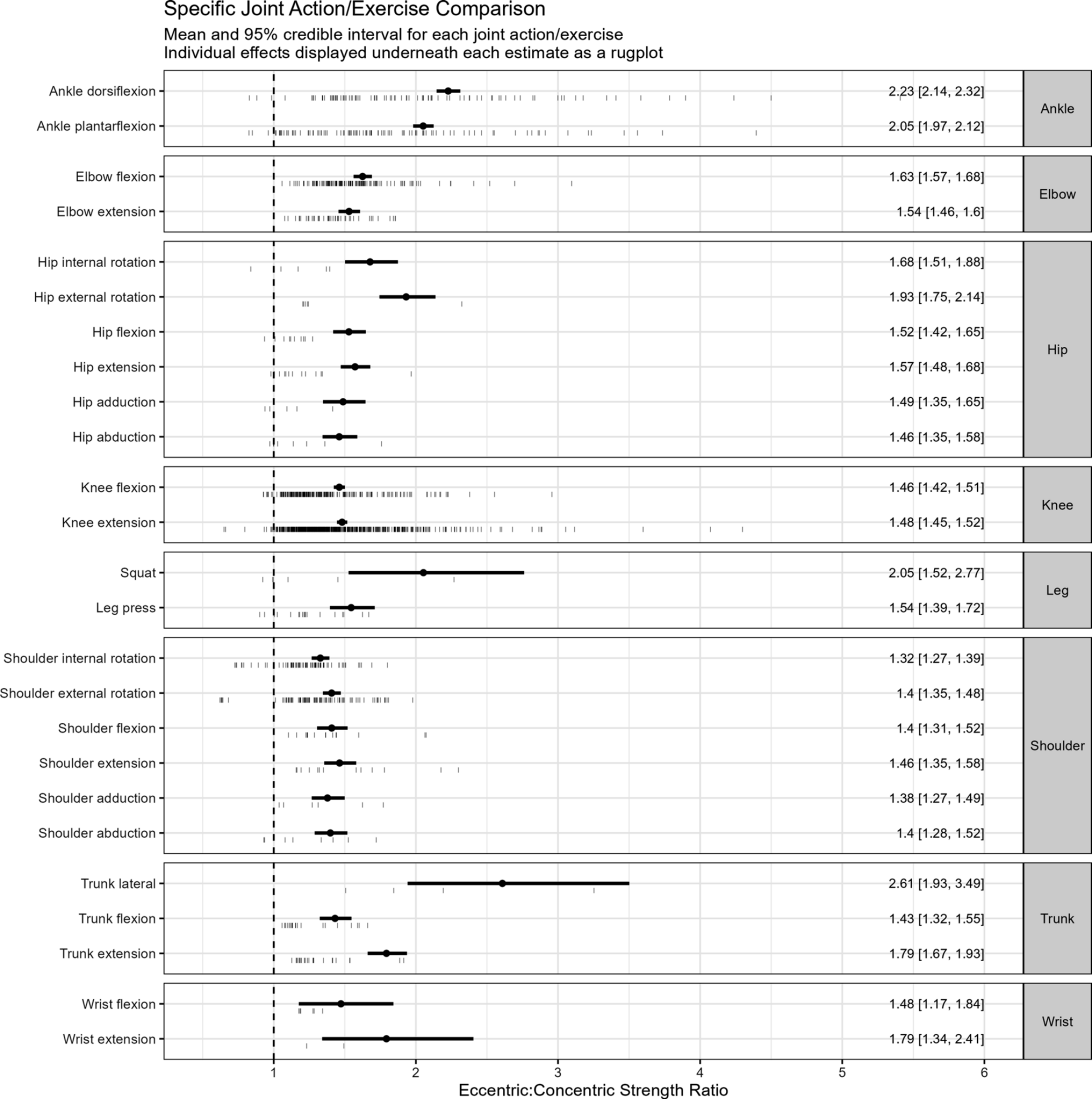
Eccentric:concentric strength ratios by joint action/exercise. Mean and 95% credible intervals are shown as the black circle and connected horizonal lines, respectively, with individual effects displayed as vertical dashes below each estimate as a rugplot. Means have been adjusted for age and movement velocity, and the individual effects are differentially weighted based on their inverse sampling variance. Thus, some means might appear to fall outside of the individual effects

**Fig. 4:**
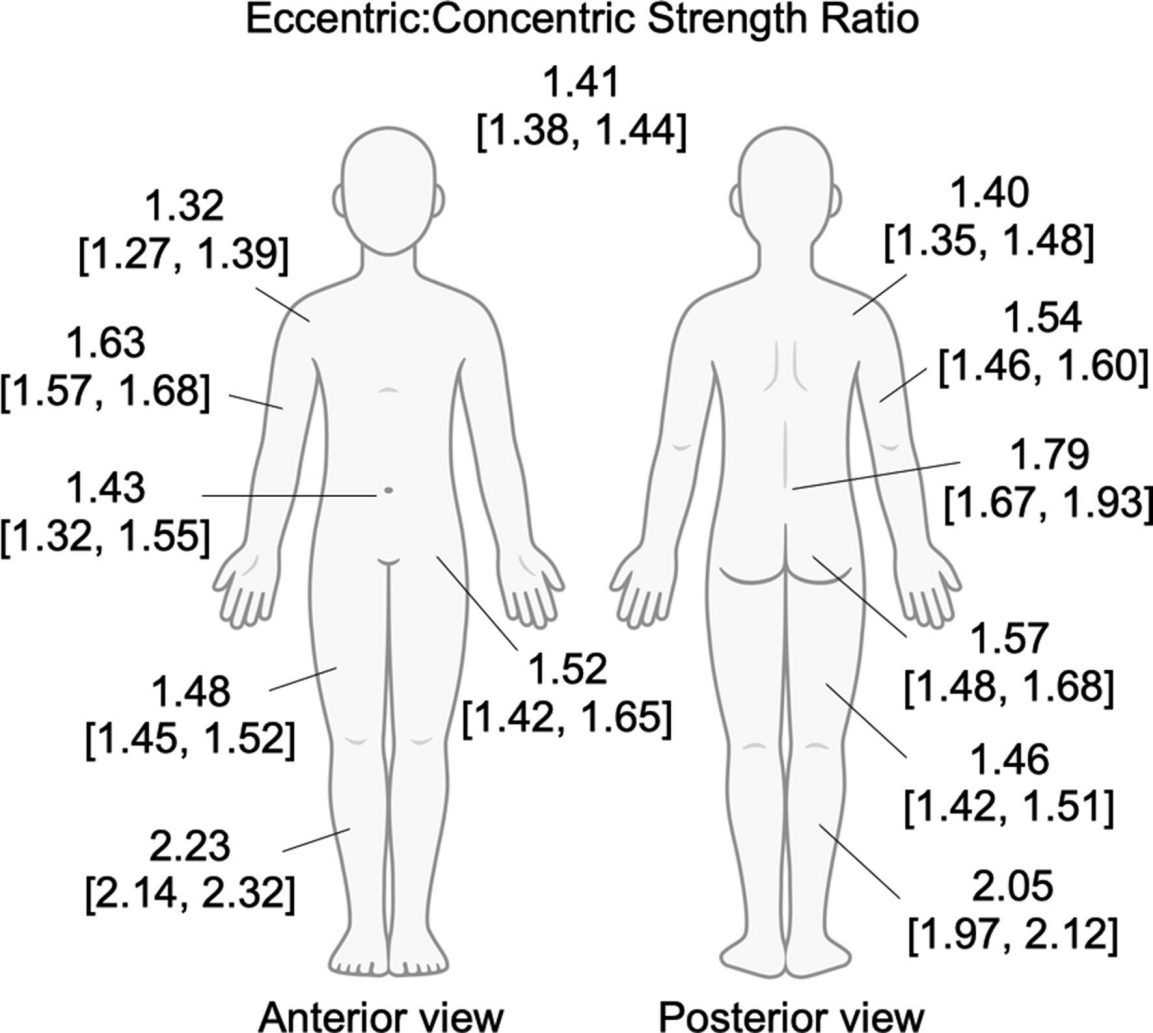
Body chart of mean and 95% credible intervals of the eccentric:concentric strength ratios by muscle group. Ratios listed for the anterior and posterior shoulder are for internal and external shoulder rotation, respectively. Ratios listed for the anterior and posterior hip are for hip flexion and extension, respectively. Ratios for all joint actions/exercises, and the individual effects analysed, are presented in Fig. 3. The body chart was obtained from Adobe Stock (https://stock.adobe.com/au)

**Fig. 5:**
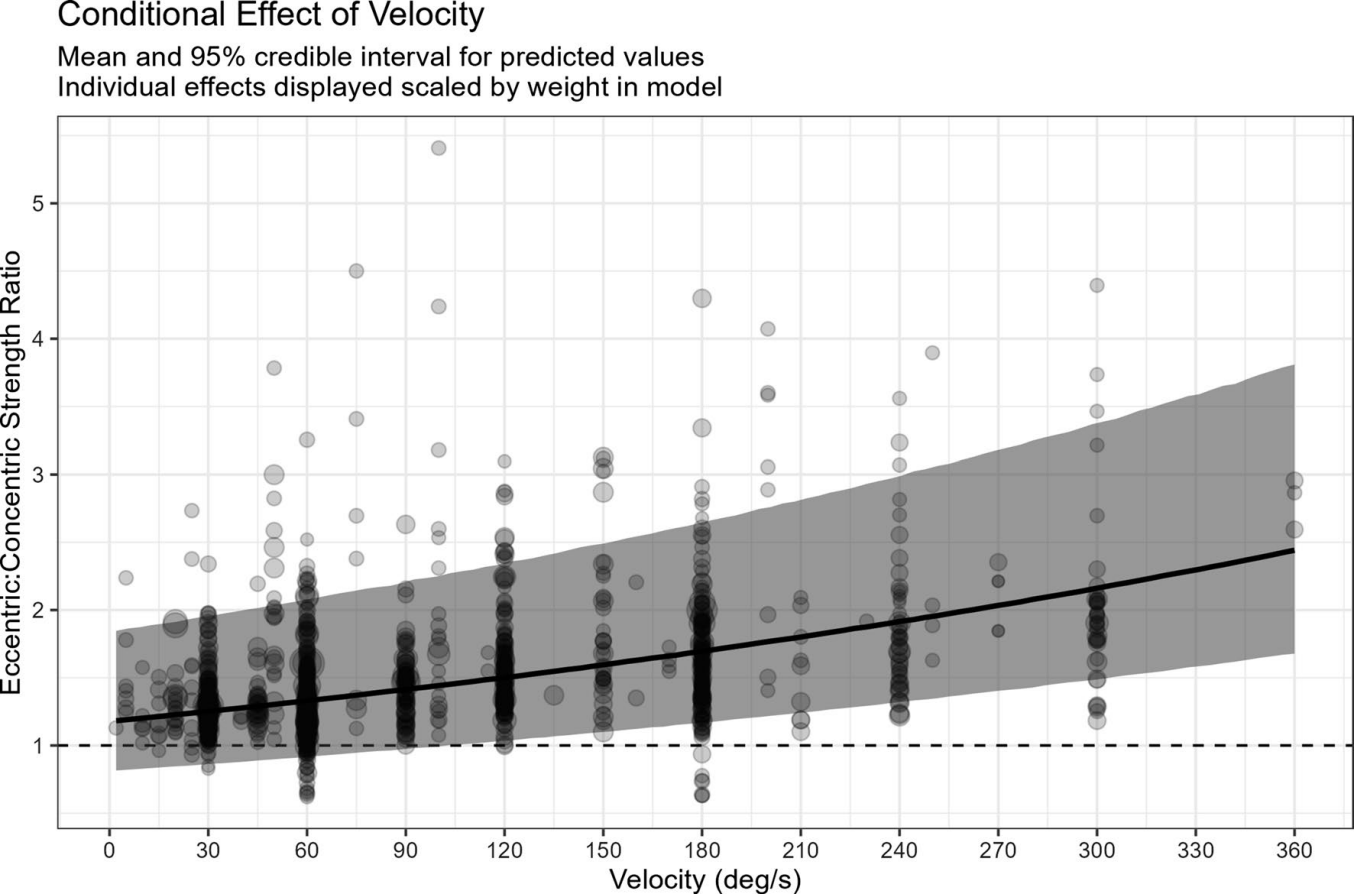
Eccentric:concentric strength ratios by test velocity. Mean and 95% credible intervals are shown as the black line and grey shaded area, respectively, with individual effects as circles with the sizes of the circles scaled to weighting in the model

Estimates of ECC:CON strength ratios by sex, age group, upper- versus lower-body joint actions/exercises, and velocity are presented in Table 1. The ECC:CON ratio was greater in older adults (1.62 [95% CI 1.57–1.68]) than younger adults (1.39 [95% CI 1.36–1.42]) and was slightly lower in men (1.38 [95% CI 1.34–1.41]) than women (1.47 [95% CI 1.43–1.51]).Whilst in general there was little difference in the ECC:CON strength ratio between upper-body (1.42 [95% CI 1.38–1.46]) and lower-body joint actions/exercises (1.40 [95% CI 1.37–1.44]), there did appear to be some heterogeneity between joint action/exercise effects on the ECC:CON strength ratio from our exploratory model (Figs. 3, 4). The number of effects in the exploratory joint action/exercise model was 1390 across 502 groups from 300 studies. However, estimates were imprecise for some joint actions/exercises (e.g. squat, trunk lateral flexion, hip internal and external rotators, and both wrist flexors and extensors). There was a clear log-linear relationship with velocity of movement, where ECC:CON increased by 0.20% for every 1°/s increase in velocity (Fig. 5).

Due to the sex and age differences in ECC:CON strength ratios, we also examined the standardized mean differences and log ratio of means for ECC and CON separately between these groups for studies that included either both men and women or both younger and older adults. This analysis examined whether differences in the ECC:CON ratio between these groups were due primarily to differences in ECC or CON strength. The summary of estimates from these models is included in the electronic supplementary materials (see https://osf.io/r7g3v). The difference between men and women was slightly greater for CON than ECC strength, favouring men, and thus leading to the slightly lower ECC:CON strength ratios among men than women. The difference between younger and older adults was larger for CON than ECC strength, favouring younger adults, and thus leading to the lower ECC:CON strength ratios among younger than older adults.

## Discussion

The purpose of this meta-analysis was to determine the magnitude of the ECC:CON strength ratio of human skeletal muscle in vivo and explore if sex, age, joint actions/exercises, and movement velocity impact it. We found consistent evidence that ECC strength is greater than CON strength. Across 335 studies, the main model estimate for the ECC:CON strength ratio was 1.41. Thus, ECC muscle strength is generally ~ 40% greater than CON muscle strength. However, the ECC:CON strength ratio is impacted by movement velocity and age and to a lesser extent sex. No difference in the ECC:CON strength ratio was observed between upper-body and lower-body joint actions/exercises generally speaking, but exploratory analysis suggested heterogeneity in the ECC:CON strength ratio across specific joint actions/exercises.

### Sex

The ECC:CON strength ratio was slightly greater in women (1.47) than men (1.38). The reason for this slight sex difference appears to be that the magnitude of the sex difference in muscle strength is greater in CON than ECC muscle actions [35]. Indeed, exploratory analysis revealed this to be true (see https://osf.io/r7g3v). One explanation for this result might be that men participate in muscle-strengthening activities more regularly than women [35]. Such activities typically involve lifting a constant load, and this load will represent a greater percentage of the CON than ECC 1RM. This might then provide a disproportionately greater stimulus for increasing CON than ECC muscle strength among men than women. A potential practical implication of this finding is that if an exercise professional prescribes ECC overload as percentage of the CON 1RM, then the multiplication factor for this computation might need to be slightly higher for women than men.

### Age

The ECC:CON strength ratio was greater in older adults (1.62) than younger adults (1.39). The likely reason for this result is that ECC strength is better preserved with aging than is CON strength [7, 50, 51]. Our exploratory analysis also revealed this to be true (see https://osf.io/r7g3v). The cause of greater ECC than CON strength preservation with aging is not completely understood, but neurological, mechanical, and cellular mechanisms could all contribute (e.g. decreased activation of agonist muscles and increased activation of antagonists muscles during CON contractions; increased connective tissue and passive and active muscle stiffness, which preserves ECC strength) (see [51] for review). Also, as aging research usually involves examination of CON rather than ECC strength, this helps to explain why, in such cross-sectional and longitudinal research, men exhibit relatively greater reductions in strength [52, 53]. In one longitudinal study of older adults, reductions in CON strength of muscles about the elbow were 2% per decade in women but 12% per decade in men [52]. A potential practical implication of this finding is that if an exercise professional chooses to prescribe ECC overload as a percentage of the CON 1RM, then the multiplication factor for this computation might need to be higher for older adults than younger adults.

### Joint Action/Exercise

In the current analysis, muscle strength measurements acquired from joint actions/exercises about the wrist, elbow, and shoulder were combined into one upper-body ECC:CON strength ratio. Similarly, strength measures acquired from joint actions/exercises about the ankle, knee, and hip were combined into one lower-body ECC:CON strength ratio. The ECC:CON strength ratio was generally similar between the upper-body (1.42) and lower-body (1.40). However, exploratory analysis revealed heterogeneity between some joint actions/exercises. We consider this analysis exploratory, in part, because of a relative lack of ECC versus CON strength data for some joint actions/exercises. Indeed, this is reflected in the imprecision in estimates for joint actions/exercises such as the squat, trunk lateral flexion, hip internal and external rotation, and wrist flexion and extension. The knee extension was the joint action/exercise studied most frequently, with 566 effects, and this was more than double the next most frequently studied joint action/exercise (i.e. knee flexors, 218 effects) (Table 2). A small number of studies also tested other exercises such as the bench press, chest press, military press, lat pulldown, and seated row; however, these were not included in the exploratory model because either only a single study included one of the exercises or velocity of movement was not reported and thus did not allow for velocity adjustment. Nevertheless, heterogeneity in the ECC:CON strength ratio between specific joint actions/exercises appears to exist. Future research should systematically explore different joint actions/exercises with large samples to obtain more precise estimates of their ECC:CON ratios. Moreover, 98% of ECC:CON strength ratios came from tests of isokinetic muscle strength, with few researchers attempting to measure ECC and CON 1RMs with free weights, weight stack machines, or plate-loaded machines. The ECC 1RM is often impractical to examine given designs of most resistance exercise equipment. However, emerging resistance exercise technologies [17, 22] could make evaluation of maximal ECC strength safer and more feasible in coming years. Such machines might then be used to establish ECC:CON muscle strength ratios for various joint actions/exercises.

**Table 2:** Number of effects for each joint action/exercise

Joint action/exercise	No. effects
Knee extension	566
Knee flexion	218
Elbow flexion	129
Ankle plantarflexion	100
Ankle dorsiflexion	90
Shoulder external rotation	90
Shoulder internal rotation	76
Elbow extension	42
Trunk extension	23
Trunk flexion	20
Leg press	18
Shoulder flexion	15
Hip extension	13
Shoulder extension	13
Hip flexion	10
Hip abduction	8
Shoulder abduction	8
Hip external rotation	6
Shoulder adduction	6
Squat	6
Wrist flexion	6
Hip adduction	5
Hip internal rotation	5
Trunk lateral	4
Wrist extension	2

### Velocity

The factor that impacted the ECC:CON strength ratio the most was movement velocity. The ECC:CON strength ratio was largest at fast velocities and smallest at slow velocities. The larger ECC:CON strength ratio at faster velocities is mostly due to the substantial reduction in CON torque that occurs as velocity increases. The mechanisms that underlie the CON and ECC force–velocity relationships of skeletal muscle are still being explored and debated [54–57]. The CON force–velocity relationship is thought to be impacted by cross-bridge kinetics (i.e. time-dependent cross-bridge attachment and detachment), neural activation, and muscle architecture [54, 55]. Viscoelastic properties of non-cross-bridge elements (e.g. titin) also appear to impact the ECC force–velocity relationship [56]. Irrespective of the mechanisms involved, our analysis revealed a log-linear relationship between test velocity and the ECC:CON ratio such that the ratio increased 0.20% for every 1°/s increase in velocity. A potential practical implication of this finding is that resistance exercise technologies that can control ECC and CON phase velocities independently can account for such differences to optimize force generation during the ECC and CON phases.

### Implications

Historically, ECC resistance exercise has been difficult to prescribe because of limitations of free weights and weight machines. ‘Releasers’, which dispose of a proportion of the ECC load after the ECC phase, have been used with free weights and weight machines to overcome such limitations [18]. However, ‘releasers’ can be difficult to use beyond the first repetition. The lack of feasibility in implementing ECC resistance exercise with such equipment explains why, in the current meta-analysis, so few studies assessed ECC 1RMs, i.e. isoinertial testing. It also explains why, in one survey, 23% of strength and conditioning coaches said inadequate equipment was the most significant barrier to implementation of ECC resistance exercise [58]. In a different survey, 57% of coaches who had never prescribed ECC resistance exercise said the main reason was ‘equipment access’ [20]. Nevertheless, new exercise technologies have the potential to make ECC resistance exercise more accessible, feasible, and safer, and feasible. Examples of such equipment include connected adaptive resistance exercise machines [22, 59, 60], flywheels [61], and motorized isokinetic devices [62]. Other ECC resistance exercise machines also exist and have been reviewed elsewhere [17]. With such equipment, independent load prescriptions for the ECC and CON phases are sometimes possible. Thus, knowledge of ECC:CON strength ratios might be useful for coaches who use such equipment to prescribe ECC overload. Currently, coaches [20] and researchers prescribe ECC loads ranging from 1.05 to 1.5 times the CON 1RM [24–33]. Results from the current analysis suggest that factors such as velocity, joint action/exercise, age, and, to a lesser extent, sex warrant consideration when determining how much ECC overload to prescribe to healthy individuals. For example, if ECC overload is computed based on the CON 1RM, then higher multiplication factors are likely necessary for older adults compared to younger adults and for faster velocities compared to slower velocities. New exercise technologies have potential to allow for isokinetic exercise and independent control of ECC and CON resistances in non-laboratory environments. Isokinetic modes in such machines might account for the impact of velocity on force. To allow participants to generate their greatest CON forces, slow movement velocities would be necessary. For the ECC phase, more leniency could be provided, as force output in the ECC phase is less impacted by velocity.

A notable limitation of the current meta-analysis is that the vast majority of studies reported ECC and CON strength from isokinetic dynamometers, whereas most strength and conditioning coaches use isoinertial equipment when prescribing ECC resistance exercise to athletes [19, 20, 23]. ECC:CON strength ratios from isokinetic dynamometry might differ from those acquired from isoinertial tests. We did not explore this potential difference in the current meta-analysis because of the relative lack of ECC:CON strength ratios from isoinertial tests. Therefore, some degree of caution is advised when attempting to apply results from the current meta-analysis to isoinertial training methods.

## Conclusion

Researchers have known for many decades that ECC strength is greater than CON strength. However, prior to the current research, the magnitude of this strength difference, and the factors that impact it, had never been submitted to meta-analysis. We report a main model estimate for the ECC:CON strength ratio of 1.41. However, the ratio is higher at faster than slower movement velocities and in older adults than younger adults. The ratio is also slightly higher in women than men. The ratio does not differ between upper- and lower-body muscles generally speaking, but an exploratory analysis indicated that there is likely heterogeneity in ratios across different joint actions/exercises. Further systematic study will be necessary to identify more precise estimates of exercise-specific ECC:CON strength ratios. Exercise practitioners can use the ECC:CON ratios from the current analysis to guide prescriptions of ECC overload to healthy individuals.

## Footnotes

1 *C –* 1, where *C* was the number of cores available on the computer used to run the analysis (build available here: https://uk.pcpartpicker.com/list/C6VXRT).

## Declarations

### Data and code availability

The extracted data, and the code used to analyse that data, are available as supplementary materials at the Open Science Framework (https://osf.io/8vt9h/).
